# Chronic Hepatitis C: Conspectus of immunological events in the course of fibrosis evolution

**DOI:** 10.1371/journal.pone.0219508

**Published:** 2019-07-18

**Authors:** Dejan Baskic, Vuk Vukovic, Suzana Popovic, Danijela Jovanovic, Slobodanka Mitrovic, Predrag Djurdjevic, Dusko Avramovic, Aleksandra Arsovic, Dragic Bankovic, Jelena Cukic, Zeljko Mijailovic

**Affiliations:** 1 Center for Molecular Medicine and Stem Cell Research, Faculty of Medical Sciences, University of Kragujevac, Kragujevac, Serbia; 2 Garrison Clinic, Kragujevac, Serbia; 3 Department of Hematology, Clinical Center Kragujevac, Kragujevac, Serbia; 4 Centar for Pathological Anatomy, Faculty of Medical Sciences, University of Kragujevac, Kragujevac, Serbia; 5 Department of Gastroenterology, Clinical Center „Dr Dragisa Misovic“, Beograd, Serbia; 6 Institute of Medical Microbiology and Immunology, Medical Faculty, University of Pristina, Kosovska Mitrovica, Serbia; 7 Department of Mathematics and Informatics, Faculty of Science, University of Kragujevac, Kragujevac, Serbia; 8 Public Health Institute, Kragujevac, Serbia; 9 Department of Infectious Diseases, Clinical Center Kragujevac, Kragujevac, Serbia; Central University of Tamil Nadu, INDIA

## Abstract

In chronically infected HCV patients emergence and evolution of fibrosis, as a consequence of virus persistence, can be considered as an indicator of disease advancement. Therefore the aim of this study was to correlate alterations of immune response in chronic HCV patients with liver histopathology. Sera cytokine levels and frequency of circulating and liver infiltrating cells were evaluated using 13plex Kit Flow Cytomix, flow cytometry and immunohistochemistry. We found that the number of circulating T lymphocytes (including CD4+, CD8+ and Treg) and B lymphocytes, as well as DCs, was higher in patients with no fibrosis than in healthy subjects. In patients with fibrosis frequency of these cells decreased, and contrarily, in the liver, number of T and B lymphocytes gradually increased with fibrosis. Importantly, in patients with advanced fibrosis, liver infiltrating regulatory T cells and DC-SIGN+ mononuclear cells with immunosuppressive and wound-healing effector functions were abundantly present. Cytokine profiling showed predominance of proinflammatory cytokines in patients with no fibrosis and a tendency of decline in level of all cytokines with severity of liver injury. Lower but sustained IL-4 production refers to Th2 predominance in higher stages of fibrosis. Altogether, our results reveal graduall alterations of immunological parameters during fibrosis evolution and illustrate the course of immunological events through disease progression.

## Introduction

Hepatitis C virus represents a major medical, social and economic problem worldwide, infecting about 3% of the world's population [1]. After acute infection about 80% of patients develop a chronic, life-long disease [2]. In approximately 20% of patients, persistent viremia leads to fibrosis, cirrhosis and hepatocellular carcinoma [3]. In chronically infected patients the rate of disease progression varies and is unique for every patient. There are many factors, viral and hosts that influence disease progression, such as genotype [4] and genetic variability of HCV virus [5], ethnicity, age and sex of patients, the strength of cellular immune response [6] and cytokine production in host [7], genetic and metabolic features of patients and coinfections with other viruses [8, 9].

During acute HCV infection coordinated action of both innate and adaptive immunity, particularly strong pathogen-specific cellular immune response, can lead to resolution of infection. However, HCV possesses delicate mechanisms for avoiding both arms of host defense. As a result, in chronic HCV infection immune response is altered, changing over time and progression of disease is accompanied with gradual deterioration of the immune response.

In our previous work we have analyzed cytokine pattern in group of chronically infected HCV patients [10] and correlated data with viral and host factors that influence or reflect progression of the disease, i.e. HCV genotype, HCV RNA load, ALT/AST ratio and the stage of fibrosis. Although statistical analysis showed no correlation between each of single parameters with cytokine levels, there were obvious differences inside every group. Therefore, in order to illustrate a pattern of immunological events through the development of disease, we decided to opt for one criteria, namely hepatic fibrosis.

In chronic HCV infection continuous liver damage and chronic inflammation induce wound-healing response. Destruction of hepatocytes and proinflammatory and profibrotic cytokines environment (TNF-α, TGF-β, IL-6) activate hepatic stellate cells, myofibroblasts and fibroblasts, which results in deposition of extracellular matrix, including fibrillar collagen, i.e. fibrosis [11]. Disease progression and massive fibrosis leads to cirrhosis. Therefore, emergence of fibrosis and fibrosis evolution, as a consequence of virus persistence, progressive inflammation and lasting immune response, can be considered as indicator of disease advancement. Thus, in order to depict alterations of immune response through the course of disease, we examined the frequency of circulating and liver infiltrating cells of innate and adaptive immunity and cytokine profile in chronically HCV infected patients and correlated data with liver histopathology. Although in some studies data are grouped according to presence or absence of fibrosis, in our opinion ranking according to stage of fibrosis can better delineate occurrences in ongoing chronic HCV infection.

## Results

The characteristics of patients participating in study are presented in Table 1. Liver biopsy specimens were scored according to Knodell and the degree of fibrosis was evaluated as no fibrosis (F0) in 7 of 24 patients (29%), F1 stage in 9 (38%) and F3 stage in 8 (33%) patients.

**Table 1:** Basic characteristics of patients and control subjects.

Characteristics	Patients	Controls
Age (years)	44,0±13,2	46,0±12,5
Male/female (N)	14/10	8/8
HCV RNA titer (copies/ml x 10^6^)	9,22±13,18	-
AST (IU/l)	127,71±39,97	22,37±5,35
ALT (IU/l)	158,63±52,38	19,31±7,85
HCV genotype N (%) -1 -3	18 (75) 6(25)	-
Knodell fibrosis N (%) -0 -1 -3	7(29) 9 (38) 8 (33)	-

### Cytokine levels decrease toward higher stages of fibrosis

**Fig 1:**
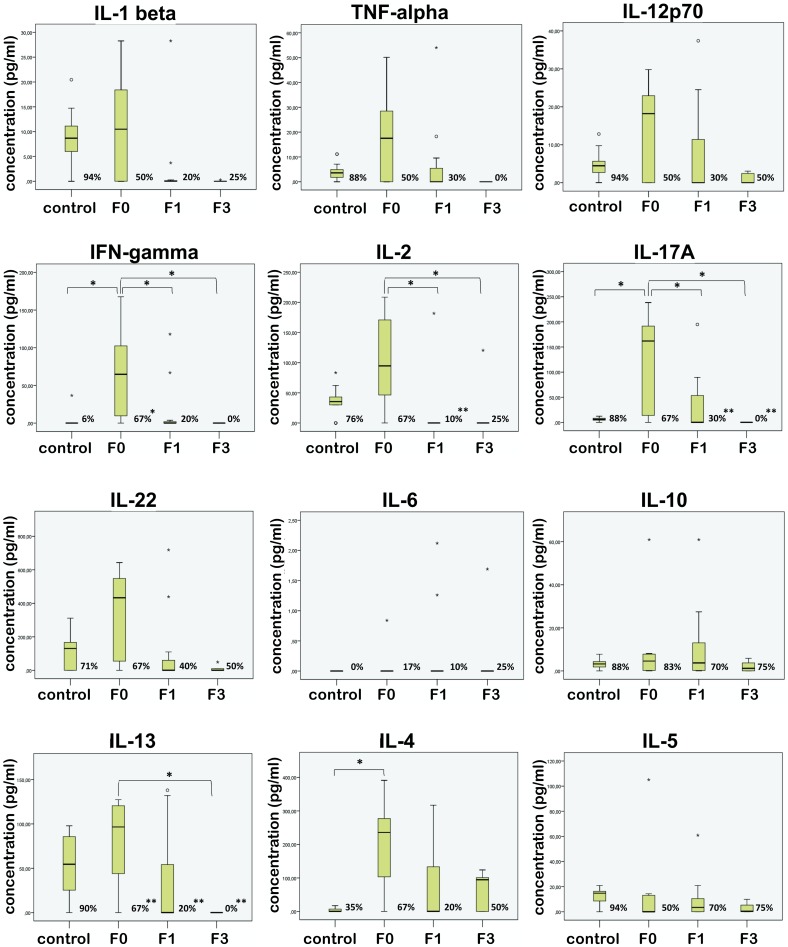
Box plots detailing sera cytokine levels in healthy controls and patients in different stages of fibrosis (F0, F1, F3). The numerals near the bars represent the percent of cytokine-producing subjects. Asterisk reflects significance of difference related to control: *p<0.05; **p<0.001.

Assembling the data in three groups, according to the stage of fibrosis (Fig 1), we showed that in chronically infected patients with no fibrosis (F0) levels of all cytokines were higher than in controls. This difference was statistically significant for Th1 cytokine IFNγ (p = 0.040), Th17 cytokine IL-17 (p = 0.031) and Th2 cytokine IL-4 (p = 0.014). Furthermore, in patients with no fibrosis (F0) levels of all cytokines, except IL-5, were higher than in patients with fibrosis and a tendency of decrease in cytokine levels toward higher stages of fibrosis was noted. In relation to cytokine levels in F0 group, statistically significant decrease in group of patients with fibrosis (F1, F3) was recorded for IFNγ (p = 0.039), IL-2 (p = 0.033), IL-17 (p = 0.039) and IL-13 (p = 0.047). Also, significantly less number of patients with fibrosis produced measurable levels of IFNγ (p = 0.007), IL-2 (p = 0.001), IL-17 (p = 0.001) and IL-13 (p<0.0005) (percent of cytokine-producing subjects are presented in Fig 1 as the numerals near the bars). Sera level of IL-9 was above detection limit only in one patient, therefore this cytokine was not further discussed.

**Fig 2:**
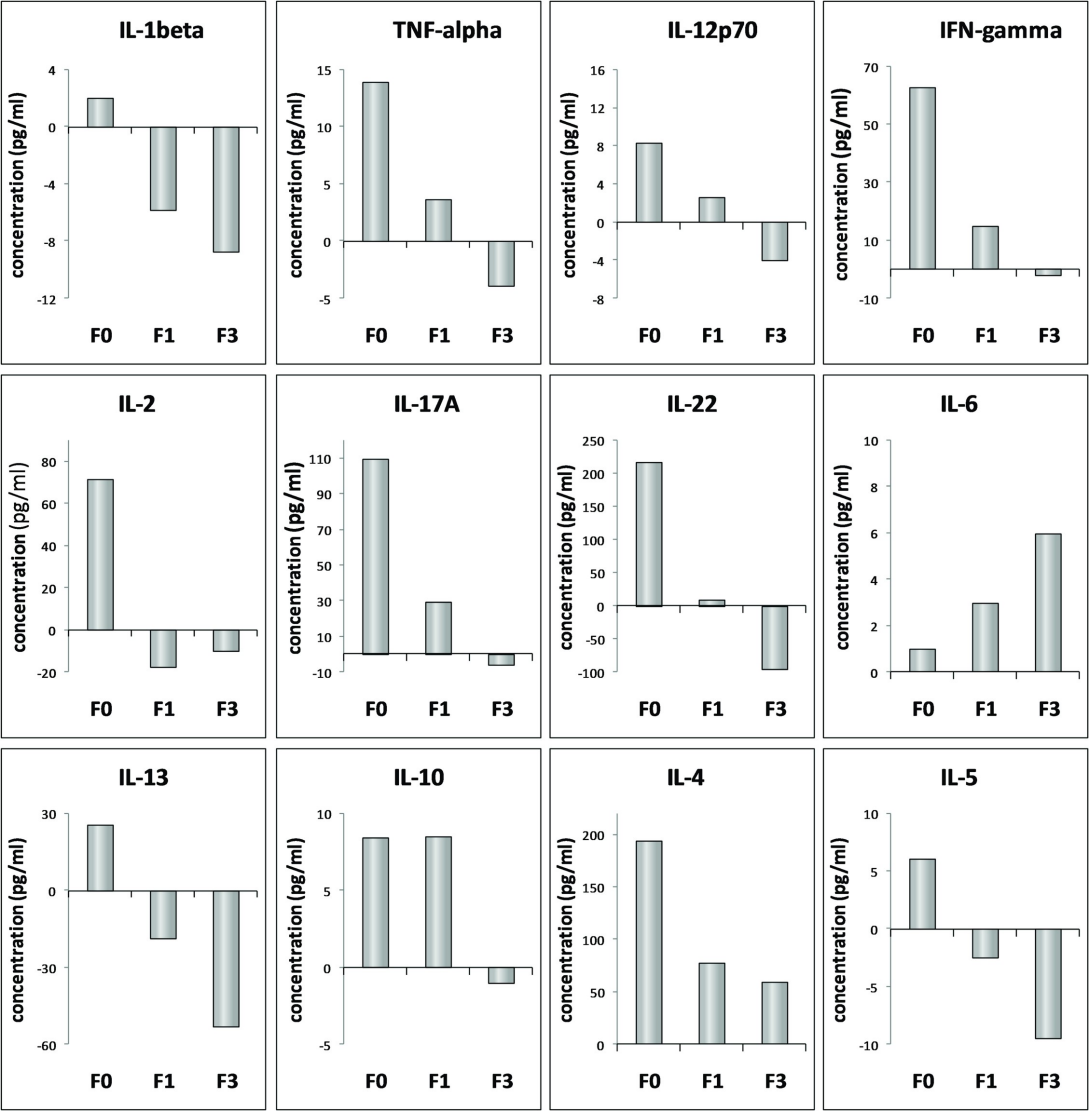
Change of cytokine profile depending on the stage of fibrosis. The bars represent change (increase or decrease) in concentration of respective cytokines in patients (F0, F1, F3) in relation to control. X-axis corresponds to no difference.

In order to illustrate changes of cytokine production pattern in the course of fibrosis evolution, difference in cytokine levels between respective stages of fibrosis (F0, F1, F3) and control are presented in Fig 2. Summarized, in comparison to control, group of patients with no fibrosis had elevated levels of all cytokines, with significantly elevated levels of IFNγ, IL-2, IL-17A, IL-22 and IL-4. In F1 patients IL-6, contrary to all other cytokines, was higher, IL-10 stayed elevated, whereas levels of other cytokines were lower comparing to F0, but the majority of them still above control. In F3 group decline in cytokine levels continued. Although lower than in F0 and F1, IL-4 remained higher than in control group. All other cytokines, except IL-6, were lower than in control. Opposite to other cytokines, value of IL-6 increased through the higher stages of fibrosis.

### The frequency of peripheral blood lymphocytes and DCs decrease toward higher stages of fibrosis

**Fig 3:**
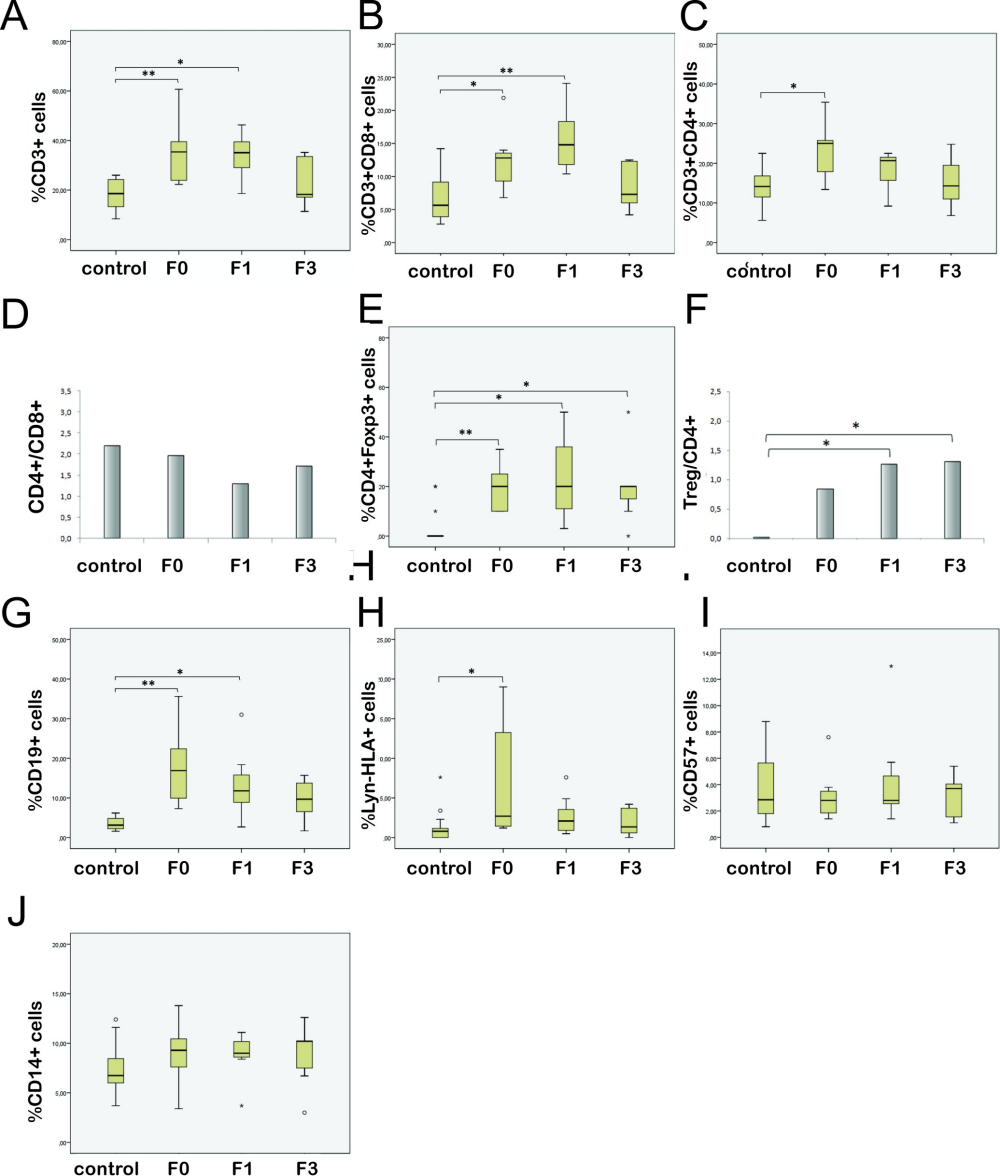
Flow cytometric analysis of peripheral blood mononuclear cells in healthy controls and patients in different stages of fibrosis (F0, F1, F3). The box plots present percentage of respective PBMNC populations in controls and patients: (A) total T lymphocytes, (B) cytotoxic, (C) helper and (E) regulatory T lymphocytes, (G) B lymphocytes, (H) dendritic cells, (I) NK cells and (J) CD14+ cells. The bar graphs show average CD4/CD8 ratio (D) and the percent of Treg cells in CD4+ population (F). Asterisk reflects significance of difference related to control: *p<0.05; **p<0.001.

Flow cytometric analysis showed that in comparison to control group, F0 patients had significantly higher percent of total T lymphocytes (CD3+, p<0.0005), helper T lymphocytes (Th) (CD3+CD4+, p = 0.011), cytotoxic T lymphocytes (CTL) (CD3+CD8+, p = 0.011), regulatory T lymphocytes (Treg) (CD3+CD4+Foxp3+, p = 0.001), B lymphocytes (CD19+, p<0.0005) and dendritic cells (Lyn-HLA-DR+, p = 0.018). Comparing to control, percentage of overall T cells (Fig 3A) and CTLs (Fig 3B) stayed significantly higher in F1 group (p = 0.004 and p = 0.001 respectively), but as well as Th cells (Fig 3C), decreased through the higher stages of fibrosis. Still, the difference in CD4/CD8 ratio between control and patients in different stages of fibrosis was not statistically significant (Fig 3D). CD3+CD4+Foxp3 regulatory T cells (Treg), barely detected in control subjects, were notably present in peripheral blood of HCV patients (Fig 3E). The percent of Treg cells in all stages of fibrosis remains higher than in control subjects (F1: p = 0.002, F3: p = 0.012). Of note, the portion of Treg cells in CD4+ population was higher in patients than in controls (p = 0.012), rise with stage of fibrosis and in F1 and F3 group was statistically higher than in control (F1: p = 0.015, F3: p = 0.010) (Fig 3F).

Percent of B cells (Fig 3G) and DCs (Fig 3H) also decreased through the stages of fibrosis, but remained higher than in controls (CD19+ control vs. F1 p = 0.006). There were no statistically significant difference in percent of CD57+ (Fig 3I) and CD14+ cells (Fig 3J) between controls and patients. Importantly, contrary to other cell populations, percent of these cells increased toward advanced fibrosis, although without statistical significance.

### Increase in number of liver infiltrating CTL, Treg and B lymphocytes and decrease of DC-SIGN expression in advanced fibrosis

**Fig 4:**
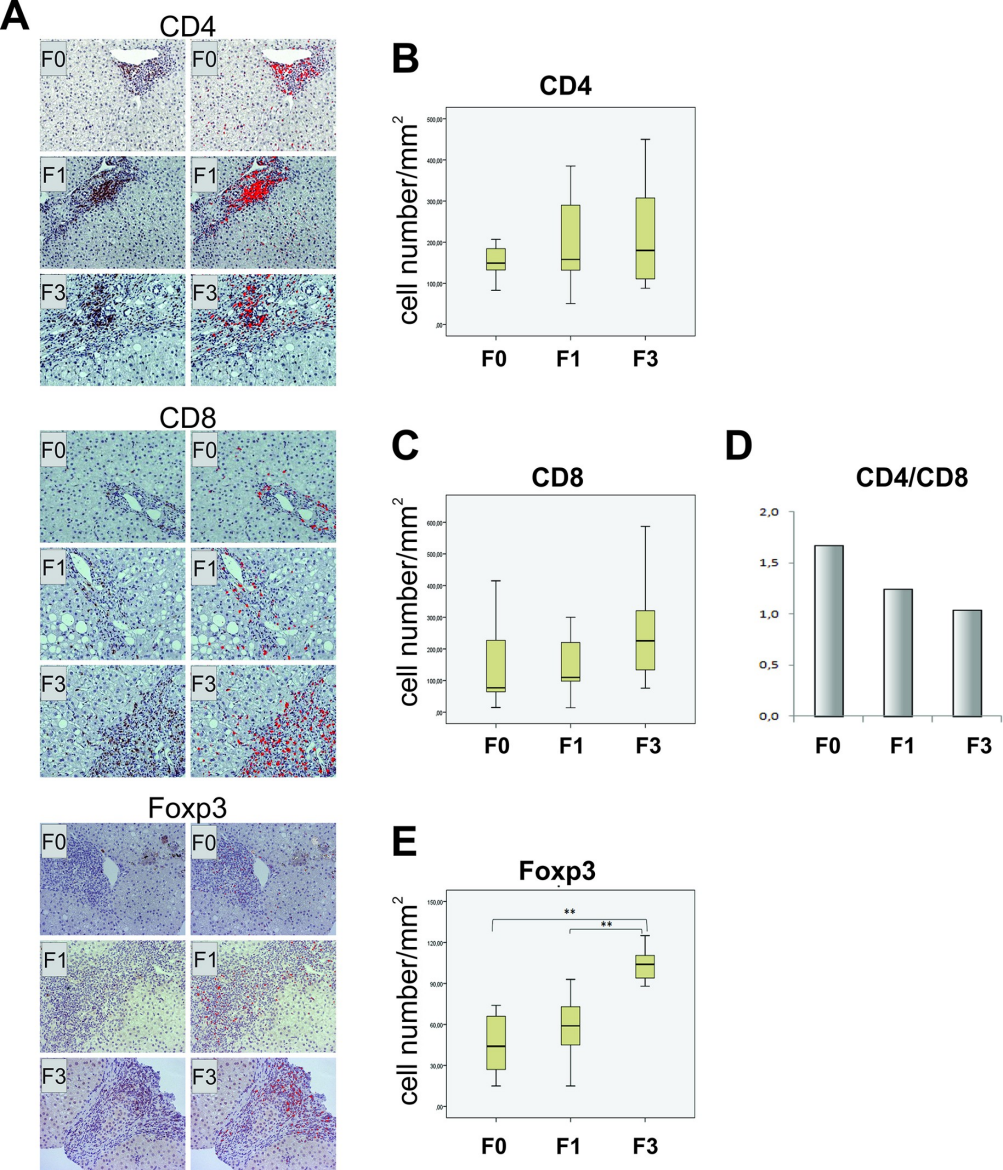
Immunohistochemical analysis of T cells in liver biopsy specimens from patients in different stages of fibrosis (F0, F1, F3). (A) Liver biopsy specimens were stained for CD4, CD8 and Foxp3, photographed, and images (left columns) were processed using ImageJ software (right columns). The results of analysis are presented by box plots as the number of positive cells per mm2 (B, C, E). The proportion of CD4 and CD8 lymphocytes is presented as CD4/CD8 ratio (D). Asterisk reflects significance of difference: **p<0.001.

To characterize and localize liver-infiltrating cells immunohistochemical analysis of liver biopsy specimens was carried out (Fig 4A). CD4+ positive Т lymphocytes were found to be predominantly localized in the portal spaces. A far fewer numbers of them was observed in the zones of confluent bridging necroses and formed septa, while rarely intralobularly, as single CD4+ Т cellular elements. There was no statistically significant difference in number of CD4+ lymphocytes ratified in different stages of fibrosis (Fig 4B).

Contrary to CD4+ cells, CD8+ T cells were more numerous intralobularly, in sinusoids and within uni and multicellular necrosis, and they were predominantly localized in bridging and periportal necrosis and portal spaces. The number of liver infiltrating CD8+ cells was higher in patients with fibrosis in comparison to F0 patients (Fig 4C), and accordantly the CD4/CD8 ratio was lower (Fig 4D).

Foxp3+ cells were found as single cell elements irregularly distributed, with dominant portal localization, while they were rarely found intralobularly within necroses and septa. Liver infiltrating Foxp3+ cells were most immanent in patients with advanced fibrosis (Fig 4E), being significantly higher than in patients without fibrosis (p<0.0005) and in F1 patients (p = 0,001).

**Fig 5:**
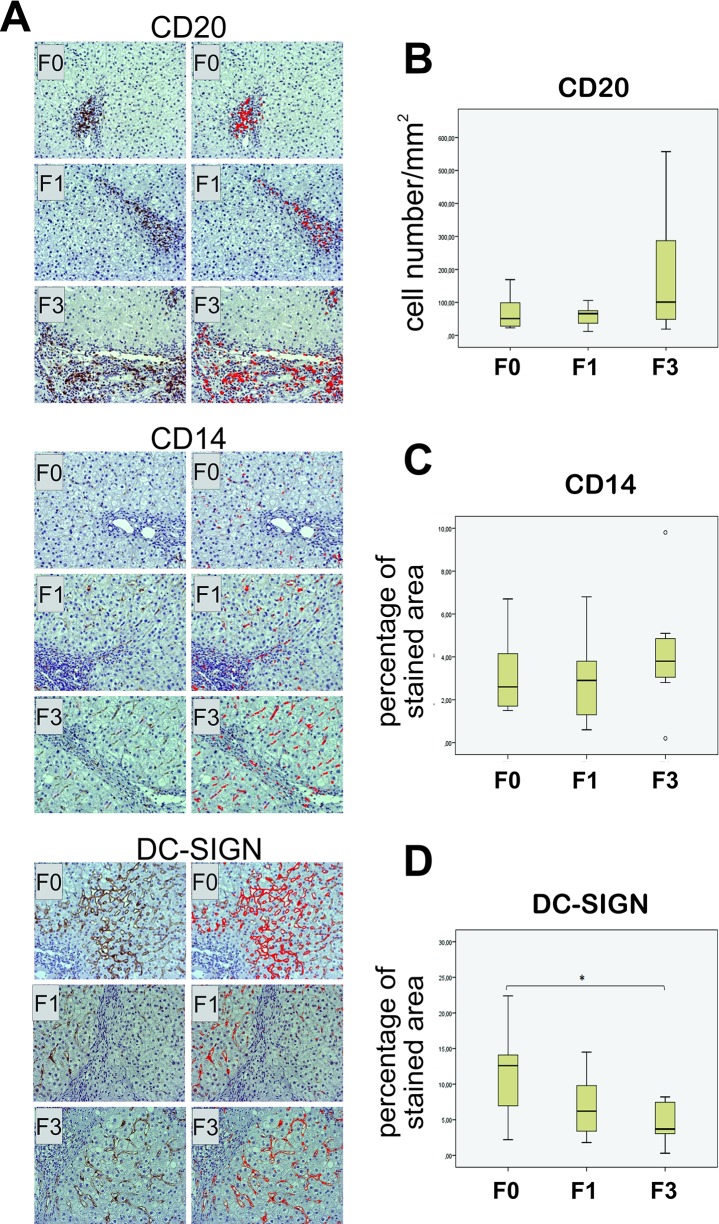
Immunohistochemical analysis of CD20+, CD14+ and DC-SIGN+ cells in liver biopsy specimens from patients in different stages of fibrosis (F0, F1, F3). (A) Liver biopsy specimens were stained for CD20, CD14 and DC-SIGN, photographed, and images (left columns) were processed using ImageJ software (right columns). The results of analysis are presented by box plots as the number of CD20+ cells per mm2 (B) or as the percent of stained area of preparation (C, D). Asterisk reflects significance of difference: *p<0.05.

Further, liver biopsy specimens were analysed for CD20+, CD14+ and DC-SIGN+ cells (Fig 5A). A large number of CD20+ В lymphocytes was found in the portal spaces and septally, while single CD20+ cells were found intralobularly. The highest number of CD20+ cells was recorded in liver biopsies of F3 patients, although there was no statistically significant differences between the groups (Fig 5B).

Expression of CD14 molecule was designated on liver sinusoidal endothelial cells and single mononuclear cells that cytologically correspond to Kupffer cells. These cells were distributed irregularly as single cells, but more often as large cell groups in the portal spaces, confluent bridging necroses, fibrous septa and rarely intralobularly in sinusoids. Because of specific distribution of CD14 molecule, its expression was presented as a percent of stained area of preparation (Fig 5C). Although this value increased with stage of fibrosis, the difference was not statistically significant.

**Fig 6:**
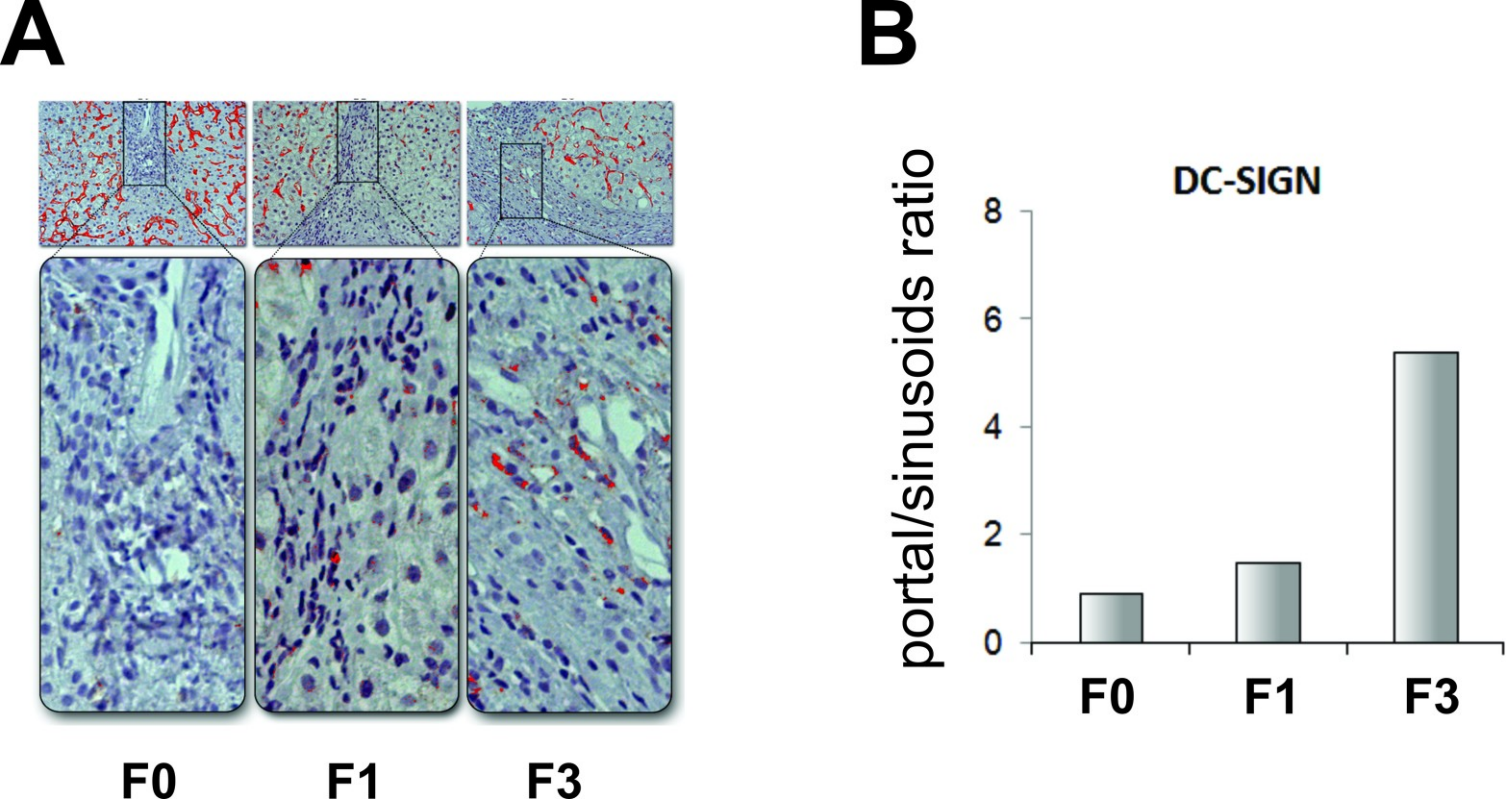
Immunohistochemical analysis of DC-SIGN+ mononuclear cells in liver biopsy specimens from patients in different stages of fibrosis (F0, F1, F3). (A) Liver biopsy specimens were stained for DC-SIGN, photographed, and images were processed using ImageJ software (upper rectangles); higher magnification of portal spaces is presented in lower rectangles. (B) The number of individual DC-SIGN+ mononuclear cells is presented as portal/sinusoids ratio (percentage of stained area in portal spaces and sinusoids).

DC-SIGN expression was observed on endothelial cells and solitary mononuclear cells. Its expression on sinusoid endothelial cells was more intense in liver parenchyma without fibrosis, especially in perivenular zone. With fibrosis progression the number of DC-SIGN+ endothelial cells and the intensity of expression decreased (Fig 5D). Consequently, the percent of stained area was the lowest in patients with advanced fibrosis (F0 vs. F3 p = 0.048). However, the number of individual DC-SIGN+ mononuclear cells, observed as single cells rarely intralobularly, and more frequently in the portal tracts and connective incomplete and complete septa (Fig 6A), increased toward higher stages of fibrosis and in F3 patients was higher than in F0 and F1 patients (Fig 6B).

## Discussion

Although a number of studies have revealed complex interactions between Hepatitis C virus and host, the data regarding immune response in chronic disease are sometimes conflicting. The origin of this inconsistency is a unique combination of viral and host factors that affects course of disease. As fibrosis evolution over time can be considered as an indicator of disease expansion, the degree of histopathological changes in the liver is, in our opinion, an adequate parameter for tracking changes in immune response during ongoing chronic infection. Therefore we grouped our data according to the stage of fibrosis.

In the first period of disease progression, in patients with no fibrosis all cytokines analyzed (except IL-5) were higher than in controls. Still, proinflammatory cytokines IFNγ and IL-17A were dominant, depicting an effort of host defense to overcome infection. Among anti-inflammatory cytokines, only IL-4 was significantly higher in F0 patients. IL-4 is cytokine that not only promotes Th2 response, but also stimulates humoral immune response and alternative activation of macrophages into M2 cells that prop wound healing and fibrosis [12].

As disease evolves hepatocellular injury, leading to fibrosis and modulation of immune response, advance. In our study development of fibrosis was associated with enduring decrease in production of all cytokines and decline in number of cytokine producing subjects and, as fibrosis progresses, the decrease was more profound. In F3 patients all cytokines, except IL-4, had lower values than controls and F0 patients, pointing to Th2 predominance in higher stages of fibrosis. IL-6 was the only cytokine whose value increased with stage of fibrosis. A number of studies have revealed that this multifunctional cytokine is increased in liver diseases with different etiologies and can be considered as indicator of hepatic dysfunction [13–16].

Persistent and efficacious cellular immune response is crucial for HCV clearance. Adaptive immune response is typically delayed in HCV infection [17] probably due to defective priming of T and B cells. A number of studies have revealed impairment of cellular immunity in chronic HCV patients as a result of failure in T cell priming, deletion of T cells in the presence of continuously high antigen level and suppression of T cell functions [18–24]. In our study we found significantly higher percent of circulating CD3+ T cells and both Th and CTL T lymphocytes in patients with no fibrosis, indicating that cellular response is not yet outworned by the virus. The percent of overall T lymphocytes and CTLs stayed high in F1 patients, but was significantly reduced in patients with advanced fibrosis. Decrease in number of circulating CTLs is probably associated with their removal to liver, where their number increased with stage of fibrosis. Percent of peripheral blood CD4+ lymphocytes significantly decreased in patients with advanced fibrosis, but the number of liver infiltrating CD4+ cells didn’t change significantly, indicating mitigation of Th response. Along with diminished cytokine production, these data point to impairment of cellular immunity.

Not only viral components, but also a subset of regulatory T cells contributes to failure of T cell-mediated immunity [25, 26]. Through cytokine secretion and via cell-to-cell contact, Tregs suppress activation and differentiation of many cell types. It has been reported that regulatory T cells restrain proliferation and induce apoptosis in activated effector T cells and block co-stimulation by DCs [27], thus hampering cellular immune response. There are opinions that Tregs have a central role in emergence of HCV persistence. In addition, certain Treg subsets stimulate fibrosis through the release of TGFβ. Recent studies have shown increased number of regulatory T cells in peripheral blood and in liver tissue of chronic HCV patients [28, 29]. Similarly, we found significantly higher number of Tregs in peripheral blood of all groups of patients in comparison to control, indicating strong suppression of immune response. Importantly, percentage ratio of these cells in CD3+CD4+ population rose toward higher stages of fibrosis and was significantly higher in F1 and F3 patients. Furthermore, the number of liver infiltrating Foxp3+ cells increases with fibrosis and in patients with advanced fibrosis was significantly higher than in F0 and F1 patients. For many years it was believed that Foxp3 is restricted to regulatory T cells. Recent studies have demonstrated Foxp3 expression in other immune cells, DCs [30], macrophages [31] and B lymphocytes [32]. These cells exert immunosupresive functions, limiting T cell proliferation and the type-1 immune response. Therefore, the data presented here indicate strengthening suppression of immune response during progression of fibrosis.

Significance of B-cell response in controlling HCV infection is currently not well defined. Although HCV-specific antibodies are detectable in serum of chronically infected patients, they are barely efficient owing to the evolution of epitope mutants [33]. As well as in other persistent viral infections, activation of humoral immune response occurs in chronic viral hepatitis, but activated B-cells are often characterized by exhausted phenotype and are therefore dysfunctional. In addition, Oliviero et al. have reported enhanced differentiation, but deficient proliferative capacity of B lymphocytes in chronic hepatitis C patients [34]. Likewise, we found that percentage of peripheral blood B cells in all groups of patients was significantly higher than in control, indicating an ongoing humoral response. Recorded decline in patients with fibrosis in comparison to F0 patients point to reduced proliferation or migration to the liver. Indeed, we found significant increase in number of liver infiltrating B cells in patients with advanced fibrosis. However, it is not clear whether those lymphocytes are recruited from peripheral blood, or they originate from lymphoid follicles in the liver, whose formation is characteristic of chronic hepatitis C [35].

Some studies have demonstrated impaired antigen presentation and production of IL-12 and TNFα by dendritic cells [36], although others failed to confirm DC dysfunction [37]. In our study we found that the percentage of circulating DCs was significantly higher in F0 patients, but in patient with fibrosis was drawn near control. Along with determined diminution of IL-12, IFNγ and TNFα sera levels proximate to higher stages of fibrosis, these data suggest diminished activation of T-cell response.

DC-SIGN is a mannose-binding lectin found to be expressed on hepatic sinusoidal endothelial cells (HESC) and on the certain subgroups of DCs and macrophages [38]. This so-called “viral attachment factor” functions as adhesion molecule for broad spectrum of pathogens, including hepatitis C virus [39]. Hepatic sinusoidal endothelial cells have unique ability to process and present captured antigens to naive T cells, inducing tolerogenic antigen-specific response and emergence of T regulatory cells [40], thus generating an immunosuppressive environment. However, in liver fibrosis of different etiologies immunogenic function of HSECs is reversed from tolerogenic to proinflammatory [41]. Yet, hepatitis C virus alters intrahepatic immunity, diminishing both innate and adaptive immune response. DC-SIGN expression is induced by Th2 cytokines IL-4 and IL-13 [42, 43]. In our study we found decrease of DC-SIGN expression on liver endothelial cells in patients with advanced fibrosis that is presumably associated with the decrease of IL-4 and IL-13 production. However, lower, but sustained IL-4 production may be in connection with increased number of single mononuclear DC-SIGN+ cells. DC-SIGN is expressed on plasmacytoid DCs and alternative M2 macrophages [35, 40]. Increased number of those cells with immunosuppressive and wound-healing effector functions [44, 45] in higher stages of fibrosis point to maintenance of tolerogenic milieu in the liver.

Presented cross-sectional study has advantages and disadvantages relative to longitudinal studies. This type of study is not time consuming, permits comparing of multiple variables at the same time and allows researcher to create theories and to prove or disprove them. However, only longitudinal studies enable reliable assesment of changes through disease progression. Still, the data presented here can be utilized to depict conspectus of immunological events in the course of fibrosis evolution.

Summarized, in the first period of chronic HCV infection, in patients with no fibrosis, the forces of immune system are recruited: circulating B lymphocytes, helper and cytotoxic T lymphocytes and dendritic cells are more numerous than in healthy subjects and proinflammatory cytokines dominate. Still, regulatory T cells are abundant causing suppression of immune response. As disease progresses, number of circulating helper T lymphocytes decreases. CTLs migrate to the site of inflammation causing more harm that results in development of fibrosis, but decreased number of circulating DCs and increased number of DC-SIGN+ mononuclear cells in the liver, along with plentiful of liver infiltrating Tregs, rule out efficient T cell struggle with the virus. The cause and the result of exhaustion of immune response is alteration of cytokine profile, from dominance of proinflammatory cytokines in patients with no fibrosis, to decline of both proinflammatory and antiinflammatory cytokines, but with pronounced IL-4 response, in patients with advanced fibrosis. Altogether, our results demonstrate graduall alterations of immune response during fibrosis evolution, illustrating possible sequence of immunological events through disease progression.

## Materials and methods

### Ethics statement

Twenty four adult patients with chronic HCV infection were recruited in Clinical center of Kragujevac, Serbia over the period of 2015 to 2017. Sixteen healthy controls, frequency matched according to the age, were selected from hospital staff. Criteria for inclusion were normal morphology and function (excretory, enzymatic and synthetic) of the liver, an absence of viral infections (HIV, HBsAg, anti-HCV) and toxic or autoimmune liver damage. Ethics Committee, Clinical Center of Kragujevac, approved the study (01-6427/5) and prior to initiation written informed consent was obtained from all subjects according to the Declaration of Helsinki.

### Study subjects

An inclusion criterion for patients was seropositivity of HCV specific antibodies and detection of HCV-RNA for at least two times 6 months apart. The liver biopsies were performed and patients were graded and staged according to Knodell et al. [46]. Patients coinfected with other hepatotropic viruses or with any possible causes of liver injury (alcohol, autoimmune diseases) were excluded from the study. None of the patients had been previously treated with immunomodulatory agents.

### Cytokine quantification

Samples were collected from treatment-naïve patients with chronic HCV infection and healthy controls as clotted blood. Sera were obtained by centrifugation. All specimens were frozen and kept at—70°C until test performing.

Circulating levels of proimflammatory cytokines IL-1β, TNFα and IL-9, Th1 cytokines IL-12p70, IFNγ and IL-2, Th17 cytokines IL-17A, IL-22 and IL-6 and Th2 cytokines IL-4, IL-5, IL-10 and IL-13 were evaluated using the 13plex Kit Flow Cytomix (eBioscience) according to manufacturer’s instructions. Data were analyzed using The FlowCytomix Pro Software.

### Flow cytometeric analysis

Whole blood samples (100 μl) were stained with anti-CD3 ECD, anti-HLA-DR PE (or ECD) (Invitrogen), anti-CD4 PE (or ECD), anti-CD8 FITC, anti-CD19 FITC (or PE), anti-CD14 FITC (or PC5), anti-CD15 PE, anti-CD57 FITC (Beckman Coulter) and isotype controls (Beckman Coulter) for 25 min. at +4°C. Intracellular staining with anti-Foxp3 (eBioscience) was performed after surface staining with anti-CD4 PE, followed by fixation and permeabilisation with Foxp3-Transcription Factor Staining Buffer Set (eBioscience) according to the manufacturer’s recommendations.

Samples were acquired on a Cytomics FC500 (Beckman Coulter) and analyzed with Flowing Software (version 2.5.1; Turku Centre for Biotechnology, University of Turku, Finland [http://www.flowingsoftware.com/]).

### Immunohistochemistry

Paraffin-embedded tissue sections (3–4μm-thick) on SuperFrost microscope slides (Thermo Scientific Gerhard Menzel) were dewaxed, rehydrated and then submitted to antigen retrieval by heating in 0,1M citrate buffer (pH 7.0) in a microwave oven for 20 min. After cooling, the slides were rinsed in distilled water for 5 min. Endogenous peroxidase was blocked by 3%H_2_O_2_ for 10 min. Slides were rinsed in dH_2_O and PBS (pH 7,5) and then incubated in a humidified chamber at room temperature with primary mouse anti-human antibodies: CD4, CD20 (Invitrogen), CD8, CD14, Foxp3 and DC-SIGN (Abcam) for 60 min. After rinsing in PBS, the slides were incubated for 30 min. with biotynilated secondary antibody. Immunodetection was performed with a Mouse HRP/DAB (ABC) Detection IHC Kit (Abcam). After 5 min. incubation, slides were washed in PBS and stained sections were counterstained with Mayer's Hematoxylin (Sigma). The slides were subsequently washed in dH_2_O and mounted in Canada Balsam. All sections were treated in parallel with an indifferent buffer to provide a negative control for each reaction.

Carl Zeiss, Axioscop 40 microscope and Canon PC 1089 camera were used for image acquisition. The pictures were taken at a magnification of 100, 200 and 400 with a resolution of 1536x2048 pixels and saved in TIFF format. Photos with magnification 400 were used for semiquantitative analysis, and high-resolution photos, with the whole specimen photographed using microscopic magnification 100 and 200, for software analysis. Data were analyzed using non-commercial application ImageJ (public domain ImageJ software17, National Institutes of Health, [http://rsb.info.nih.gov/ij]) and algorithm that enables semi-automatic or automatic analysis. The number of positive cells was estimated through the ratio of surface area with stained cells and the total area of preparation, and expressed per mm^2^ (N/mm^2^).

### Statistics

Statistical analysis was performed using the SPSS 19.0 (Statistical Package for Social Science for Windows 19).The Kolmogorov-Smirnov test was used for determination of quantitative data distribution. Comparisons of mean values between two groups were analyzed by Mann-Whitney U test or unpaired Student’s t-test as appropriate. Comparisons of mean values between n groups (n > 2) were analyzed by 1-way ANOVA. Bonferroni’s test was used for multiple comparisons. The relationship between categorical variables was examined using Fisher’s exact test.
