# Correction: Chronic Hepatitis C: Conspectus of immunological events in the course of fibrosis evolution

**DOI:** 10.1371/journal.pone.0221142

**Published:** 2019-08-08

**Authors:** Dejan Baskic, Vuk Vukovic, Suzana Popovic, Danijela Jovanovic, Slobodanka Mitrovic, Predrag Djurdjevic, Dusko Avramovic, Aleksandra Arsovic, Dragic Bankovic, Jelena Cukic, Zeljko Mijailovic

Additional affiliations are missing for the fourth, sixth, and eleventh authors. Danijela Jovanovic and Predrag Djurdjevic are also affiliated with Department of Internal Medicine, Faculty of Medical Sciences, University of Kragujevac, Kragujevac, Serbia. Zeljko Mijailovic is also affiliated with Department of Infectious Diseases, Faculty of Medical Sciences, University of Kragujevac, Kragujevac, Serbia.

## References

[1] Baskic D, Vukovic V, Popovic S, Jovanovic D, Mitrovic S, Djurdjevic P, et al (2019) Chronic Hepatitis C: Conspectus of immunological events in the course of fibrosis evolution. PLoS ONE 14(7): e0219508 10.1371/journal.pone.0219508 31318916 PMC6638930

